# How I learned to redefine academic success as a senior postdoc

**DOI:** 10.7554/eLife.83251

**Published:** 2022-10-12

**Authors:** John J Tukker

**Affiliations:** 1 German Center for Neurodegenerative Diseases (DZNE Berlin) Berlin Germany

**Keywords:** sparks of change, career, postdoc

## Abstract

After many professional twists and turns, a researcher in his forties reconsiders what it means to ‘make it’ in science.

Many young scientists, I suspect, secretly feel that they are special, and long (even more secretly) to be recognized for their uniqueness. As a child, I even dreamt about one day having a statue of myself in a park, and old friends still make fun of me for those childhood dreams of grandeur. Clearly, however, at the age of 46, I am not a famous scientist; I am not even a university professor. But while I may not have ‘reached the top’, I still find deep satisfaction in my work and life as a scientist.

I had my first brush with research during my last year studying artificial intelligence at university. Working in a lab among so many smart people, I fell for the idealism of trying to understand the brain, our humanity in a sense. I remember reading that Newton had to be reminded by his housekeeper to eat or sleep; at the time, I was deeply inspired by this obsessive way of doing science, of *living* science, which was so different from the no-nonsense Calvinistic values I had grown up with.

Soon after graduation I landed a research assistant position at an Ivy League university in the United States, and, from then on, I was completely hooked. The work was tough, but I got addicted to seeing new results, diving deeper into the literature, designing new experiments, and then doing it all over again. That feeling has never really stopped. I revelled in the late nights, the freedom, the specialness of it all. It sounds so naïve in retrospect, but I truly felt called to this path. I set out to become a professor one day.

From there, things seemed to be building to a crescendo. I attended my first conference, published my first paper, joined a prestigious academic program in Germany and then started my PhD at Oxford as “the new star of the lab”, as one cynical postdoc from Chile repeatedly called me. After a year or so, I duly produced my first paper and submitted to a high-impact journal.

It was rejected. We revised and resubmitted to an even more prominent publication. Rejected again. The paper finally ended up in a respectable but not spectacular journal. No harm done, “the editors get it wrong sometimes” was the general feeling. Much like how a certain class of wealthy people might find it vulgar to talk about money, impact factors were never openly discussed in the lab. But when my second paper ended up in the same respectable but not spectacular journal, doubts about my career prospects started to creep in. By then it was obvious to me that if I wanted to become a professor, I needed more. Still, I was confident that I would make it work, that I was good enough as a scientist to at least have a modest lab of my own one day. There was really no other option in my mind.

So, despite having had depression during my PhD, my next step was to pursue a high-risk postdoc in Berlin. Determined to finally get the ‘big’ paper I felt I needed for my career, I planned to use the most challenging and innovative approach in my field at the time. Almost five years later, I had to admit that the risk had not paid off. I had given it all I could, but the results were just not as dazzling as I had hoped. I felt lost, anxious, exhausted.

I was at a crossroads. I could use my remaining academic credit to pursue yet another high-risk opportunity, and indeed I successfully applied to two world-leading institutions in the United States. By then, though, I also had a family to consider. I spent many a night looking at how much it would cost for us to relocate to Seattle or the San Francisco Bay Area (and still do sometimes). But I just couldn’t make the numbers work. And even if we managed, as my wife believed we could, would I really be able to pour in the hours and energy that these projects would need? What would happen if I failed? Or if I were to become depressed again? In the end, I chose to stay in Berlin. I found another postdoc position in a lab run by a scientist who is not only very successful, but also genuinely nice.

For a while, I debated what my next steps should be. I changed to a more translational research direction, in an effort to be more attractive for pharmaceutical companies. I also invested a lot of time teaching anatomy to medical students, to gain credibility for possible anatomy professorships. Yet, I slowly came to realise these jobs would not make me happy. Doing fundamental research is what makes me happy.

And, by and large, that is what I do. I’m designing, setting up and performing experiments, and analyzing and discussing results (mostly those of others researchers), all driven by the same intrinsic curiosity I’ve had since I was a child. I get to supervise students, assess manuscripts, write review papers and contribute to publications. I think about exciting questions, share this excitement, communicate, connect. Of course, I also take on many organisational tasks, but I genuinely enjoy getting to improve how the lab is run.

In some sense, I am in an incredibly privileged situation. While my position has no job security and depends on third-party funding from my group leader, I have no reason to believe that this source of income will stop in the foreseeable future. I can afford to do what I love without fearing sudden unemployment. I truly hope that such ‘permanent postdoc’ positions will not remain so uncommon. It is such a waste to drive away motivated and competent scientists who, like me, can contribute to research — no matter their age or whether they are a professor. In my opinion, a postdoc is *not* a training stage, unless you consider ‘training’ any job that includes some aspect of self-development.

I am very aware that being a 40-something postdoc is not exactly glamorous; in fact, it would have sounded like a nightmare to my 30-year-old self. To be honest, the lack of status associated with the title does hurt sometimes. If you don’t have your own lab, if you’re not producing last-author papers and getting grants, you are taken less seriously as a scientist. When people discuss their posters with me at conferences, they may look around a bit more, anxious not to miss the opportunity to talk to a ‘big fish’. I vividly remember the discomfort that once flashed onto an old labmate’s face after I told her that I was *still* a postdoc. “I would have expected you to make it, of all people”, she replied. She meant it nicely, of course.

But the truth is, I did make it. Just not in the way I had planned. I found a job where I get to do things I love every day (or at least most days… science can be frustrating). In the end, that is much more important than status, or the statue that I didn’t get.

## Share your experiences

This article is a Sparks of Change column, where people around the world share moments that illustrate how research culture is or should be changing. Have an interesting story to tell? See what we’re looking for and the best ways to get in touch here.

